# Enhancement of the Aroma Compound 2-Acetyl-1-pyrroline in Thai Jasmine Rice (*Oryza sativa*) by Rhizobacteria under Salt Stress

**DOI:** 10.3390/biology10101065

**Published:** 2021-10-19

**Authors:** Kawiporn Chinachanta, Arawan Shutsrirung, Laetitia Herrmann, Didier Lesueur, Wasu Pathom-aree

**Affiliations:** 1 Doctor of Philosophy Program in Environmental Soil Science, Faculty of Agriculture, Chiang Mai University, Chiang Mai 50200, Thailand; kawiporn.ch@cmu.ac.th; 2 Department of Plant and Soil Sciences, Faculty of Agriculture, Chiang Mai University, Chiang Mai 50200, Thailand; arawan.s@cmu.ac.th; 3 Alliance of Bioversity International and Centre International of Tropical Agriculture (CIAT), Asia Hub, Common Microbial Biotechnology Platform (CMBP), Hanoi 10000, Vietnam; L.Herrmann@cgiar.org (L.H.); didier.lesueur@cirad.fr (D.L.); 4 School of Life and Environmental Sciences, Faculty of Science, Engineering and Built Environment, Deakin University, Melbourne, VIC 3125, Australia; 5 Centre de Coopération Internationale en Recherche Agronomique pour le Développement (CIRAD), UMR Eco&Sols, Hanoi 10000, Vietnam; 6 Eco&Sols, Université de Montpellier (UMR), CIRAD, Institut National de la Recherche Agricole, Alimentaire et Environnementale (INRAE), Institut de Recherche pour le Développent (IRD), Montpellier SupAgro, 34060 Montpellier, France; 7 Research Center of Microbial Diversity and Sustainable Utilization, Department of Biology, Faculty of Science, Chiang Mai University, Chiang Mai 50200, Thailand

**Keywords:** 2-acetyl-1-pyrroline, 2AP, aromatic rice, Thai jasmine rice, *Oryza sativa*, plant growth-promoting rhizobacteria, salinity stress

## Abstract

**Simple Summary:**

The major aroma compound (2-acetyl-1-pyrroline) of the world-famous Thai jasmine rice, variety KDML105, has declined due to high soil salinity and agrochemical input. In this work, the rhizobacteria from rice were investigated for the aroma compound’s production, as well as their potential for increasing the compound content in Thai jasmine rice seedlings under saline conditions. Our results provide evidence that the addition of aroma compound-producing rhizobacteria increases the aroma content in the rice seedlings under salt stress. *Sinomonas* sp. strain ORF15-23 which colonize the rice roots, is a promising rhizobacteria in promoting the aroma level of the Thai jasmine rice grown under salt stress and could be developed as a bioinoculant for Thai jasmine rice cultivation in a salt-affected area.

**Abstract:**

Thai jasmine rice (*Oryza sativa* L. KDML105), particularly from inland salt-affected areas in Thailand, is both domestically and globally valued for its unique aroma and high grain quality. The key aroma compound, 2-acetyl-1-pyrroline (2AP), has undergone a gradual degradation due to anthropogenic soil salinization driven by excessive chemical input and climate change. Here, we propose a cheaper and an ecofriendly solution to improve the 2AP levels, based on the application of plant growth-promoting rhizobacteria (PGPR). In the present study, nine PGPR isolates from rice rhizosphere were investigated for the 2AP production in liquid culture and the promotion potential for 2AP content in KDML105 rice seedlings under four NaCl concentrations (0, 50, 100, and 150 mM NaCl). The inoculation of 2AP-producing rhizobacteria resulted in an increase in 2AP content in rice seedling leaves with the maximum enhancement from *Sinomonas* sp. ORF15-23 at 50 mM NaCl (19.6 µg·kg^−1^), corresponding to a 90.2% increase as compared to the control. Scanning electron microscopy confirmed the colonization of *Sinomonas* sp. ORF15-23 in the roots of salinity-stressed KDML105 seedlings. Our results provide evidence that *Sinomonas* sp. ORF15-23 could be a promising PGPR isolate in promoting aroma level of Thai jasmine rice KDML105 under salt stress.

## 1. Introduction

The famous Thai jasmine rice (*Oryza sativa*) (Kao Dok Mali 105; KDML105) has enjoyed an international reputation for its unique aroma and premium grain quality [1]. The rice cultivar KDML105 is mainly cultivated in the northeastern region of Thailand, particularly in the Thung Kula Rong-Hai (TKR) area, which covers 2.1 million rai (1 hectare = 6.25 rai) and provides the highest grain quality with a unique aroma [2,3,4]. Aromatic rice varieties including KDML105 usually give low yield, but its premium price generates higher profit for the farmers compared to nonaromatic rice [3,4,5]. Thai jasmine rice KDML105 is considered a drought- and salt-sensitive cultivar [6,7,8]. Some environmental factors together with cultivation practices have been shown to affect rice aroma. In general, rice yield and quality are negatively affected by salinity stress [9,10,11,12,13,14]. Soil salinity with an electrical conductivity (EC) value higher than 8.0 dS·m^−1^ was reported [15,16] to reduce grain yield and boiled rice quality but increase the level of aroma compounds in the aromatic rice grains [9,17,18]. Unfortunately, recent excessive soil salinity and water shortage negatively affect both yield and grain quality of KDML105 rice, particularly levels of the aroma compound 2-acetyl-1-pyrroline (2AP) [1].

The salinization problem in agriculture, including the TKR region, is magnified by high agro-chemical inputs together with increased incidents of drought as the consequence of climate change [19,20,21]. Recently, a progressive reduction in 2AP aroma was observed due to the shift toward farming practices which rely heavily on the use of chemicals (fertilizers, herbicides, and pesticides). The overuse of chemical fertilizers is leading to a depletion in rice grain quality, particularly the aroma quality [19,22,23]. For example, the application of nitrogenous fertilizers, the use of pesticides, and usage regimes in agriculture from traditional to modern were reported as causes of aroma loss in Indian aromatic rice [22]. In addition, the best-quality Tarori basmati rice, particularly the aroma score, was achieved using 100% farmyard manure [23].

The aroma compound, 2-acetyl-1-pyrroline (2AP), has been identified as the major aromatic compound in aromatic rice, including the KDML105 cultivar, and it is recognized as a criterion for premium- or good-quality aromatic rice, thereby determining its price [24,25]. The 2AP compound in aromatic rice has been proven to synthesize from proline, a key nitrogen precursor, and it can be detected in all aboveground plant tissues of the aromatic rice plant [26]. Biosynthesis of 2AP has also been recorded in a wide range of organisms including plants and microorganisms [5,9,27,28,29]. Rice rhizobacterial strains of *Pseudomonas*, *Enterobacter*, and *Acinetobacter* were reported as producers of 2AP. These rhizobacteria, particularly high 2AP-producing isolates, increased the 2AP level in the grains of aromatic rice variety Basmati-370 [29]. Recently, *Enterobacter hormaechei* (AM122) and *Lysinibacillus xylanilyticus* (DB25) having the property of synthesizing 2AP were isolated from the rhizosphere of aromatic rice cultivars in India. The inoculation of these bacteria in consortium resulted in a significant improvement in vegetative growth, yield, and 2AP content over mono inoculation and control [5].

The application of beneficial microbes offers a sustainable alternative way to restore the rice aroma, as well as increase yield [5,29]. Our previous investigation in the TKR area indicated that much higher soil microbial populations was recorded in areas of organic rice farming (ORF) than conventional rice farming (CRF). These high microbial population in the ORF system provided higher soil organic matter content and higher total nitrogen content in the soil, as well as 3.5 times higher 2AP content in rice grains than those in the CRF system [30]. Therefore, the use of 2AP-producing PGPR, especially in the ORF system, to promote rice growth and 2AP level, as well as minimize the rice production cost, could be of interest. 

The benefits of PGPR have been widely recognized in both normal and salt-affected soil [21,31,32,33]. In general, PGPR exhibit various mechanisms to enhance plant growth, e.g., phytohormone production, biological nitrogen fixation, phosphate and potassium solubilization, and siderophore production [34]. Under salt-stress conditions, salt-tolerant PGPR (ST-PGPR) are able to help plants to enhance osmotic adjustment and detoxification of reactive oxygen species by antioxidants [21,31,33,35,36,37]. ST-PGPR are now being used as bioinoculants for enhancing crop yields, protection from phytopathogens, and improving soil health [38,39]. Growth promotion and alleviation of salt stress by ST-PGPR inoculation has been recorded in various bacterial genera, both Gram-positive and Gram-negative, e.g., *Bacillus, Pseudomonas,* and *Streptomyces* [31,32,40,41]. ST-PGPR *Bacillus nanhaiensis* strain TY0307 exhibited promising ability in promoting salt tolerance, proline accumulation, growth, and yield of rice under salt-stress conditions [41]. Suksaard et al. [42] demonstrated that *Nocardiopsis* sp. 1SM5-02, *Pseudonocardia* sp. 3WH5-01, *Streptomyces* sp. 2SH3-07, and *Streptomyces* sp. 3SH5-05, isolated from sea water and mangrove sediments, enhanced the growth of KDML105 rice seedlings under saline (100–200 mM NaCl) and non-saline conditions. Similarly, inoculation of salt-sensitive rice cultivars with *Brevibacterium linens* RS16 isolated from coastal soil significantly improved the salt tolerance and growth of salt-stressed rice (*O. sativa*) [12]. In addition, plant growth-promoting *Streptomyces* isolate S2-SC16 from mangrove was reported to not only promote the growth of the KDML105 rice seedlings but also have the ability to restrict the infection of plant fungal pathogen *Fusarium fujikuroi* [43]. Recently, salt-tolerant bacteria belonging to the genera *Bacillus*, *Exiguobacterium*, *Enterobacter*, *Lysinibacillus*, *Stenotrophomonas*, *Microbacterium*, and *Achromobacter* were able to alleviate salt stress and promote the growth of rice (*O. sativa*) seedlings in a pot experiment under greenhouse condition [44].

From the above investigations, we opine that the use of 2AP-producing ST-PGPR could benefit rice growth, as well as production of the 2AP aroma compound, under salinity stress. To the best of our knowledge, the use of 2AP-producing ST-PGPR for enhancing the aromatic quality of KDML105 rice has not been studied. In the present investigation, the previously screened PGPR isolates capable of solubilizing phosphate and potassium, as well as producing siderophore [45], were selected to investigate their 2AP production in the culture broth. All isolates were then used to inoculate *Oryza sativa* KDML105 rice seedlings to evaluate their potential to promote the production of a major aromatic compound, 2AP, in the seedlings under salt stress. We hypothesized that the inoculation of the 2AP-producing PGPR isolates obtained from the KDML105 rice rhizosphere would enhance the aroma level of this rice cultivar.

## 2. Materials and Methods

### 2.1. Rice Rhizobacterial Isolates

Nine PGPR were isolated from the rhizospheric soil of Thai jasmine rice (*Oryza sativa*) KDML105 using a conventional serial dilution and plating technique on nutrient agar. These isolates exhibited different levels of phosphate and potassium solubilization, as well as siderophore production, determined in a previous study [45] (Table 1). Quantitative determination of solubilized P and K was performed using a spectrophotometer and atomic absorption spectrophotometer, respectively. Siderophore type was determined by ferric perchlorate assay for hydroxamate type and Arnow assay for catecholate type [45]. Rhizobacterial isolates were obtained from either organic rice farming (ORF) or conventional rice farming (CRF). The five ORF strains were *Sinomonas* sp. ORF4-13, *Enterobacter* sp. ORF10-12, *Micrococcus* sp. ORF15-19, *Micrococcus* sp. ORF15-20, and *Sinomonas* sp. ORF15-23. The four CRF strains were CRF5-8 (unidentified), *Sinomonas* sp. CRF14-15, *Burkholderia* sp. CRF16-3, and *Bacillus* sp. CRF17-18.

### 2.2. Determination of 2AP Production by Rhizobacterial Isolates in Liquid Culture

One loopful of each 3 day old culture of each rhizobacterial isolate was transferred to 150 mL of nutrient broth (NB) (Hi Media, Mumbai, India) in a 500 mL Erlenmeyer flask and incubated with shaking (120 rpm) at room temperature (28–35 °C) for 7 days. The supernatant of each culture was obtained by centrifugation at 10,000 rpm for 10 min (4 °C) and primarily screened for the production of 2AP by sniffing [46]. To confirm 2AP production in the liquid culture, each positive strain (by sniffing) was further cultivated as described above in 2 L of NB medium for 7 days. After that, the 2 L culture broth was concentrated to about 200 mL using a rotary vacuum evaporator. The pH of the culture liquid was adjusted to pH 8.0 and then extracted with diethyl ether (2 × 70 mL). The diethyl ether layer was collected and concentrated to approximately 3–4 mL, using a Vigreux distillation column, and injected into a GC–MS for 2AP analysis [46]. 

### 2.3. Effect of Rhizobacterial Inoculation on 2AP Level in Rice Seedlings Grown under Salt Stress

In the present study, the ability of nine selected rhizobacterial isolates in promoting the 2AP level in KDML105 rice leaves under various concentration of NaCl was evaluated at harvesting time of 30 days after transplanting.

#### 2.3.1. Experimental Design

The experiment was conducted using a completely randomized design (CRD) in a factorial scheme (10 × 4) with three replications. In total, there were nine selected rhizobacterial isolates plus one uninoculated control (ten treatments), and four NaCl concentrations (0, 50, 100 and 150 mM NaCl). Each treatment had its corresponding control with the same concentration of NaCl and no rhizobacterial inoculation.

#### 2.3.2. Preparation of Rhizobacterial Isolates

All rhizobacterial isolates were grown in 25 mL of nutrient broth (NB) for 3 days at 37 °C with shaking at 120 rpm. The biomass of rhizobacteria was collected by centrifugation at 10,000 rpm for 15 min to separate the culture broth and the cells. Cells were suspended in 100 mL of sterile distilled water to obtain the concentration of 10^6^ colony-forming units (CFU) per mL (OD_600_~0.2). This cell suspension was used for rice seed biopriming and rice seedling inoculation. Sterile distilled water was used as a negative control (without rhizobacterial inoculation).

#### 2.3.3. Rice Seedlings

*Oryza sativa* L. KDML105 rice seeds, from the Community Enterprise of Thamo Organic Agricultural Group, Surin Province, were surface-sterilized in a mixture of 0.2% Tween-80 and 2% sodium hypochlorite for 3 min. Seeds were then washed three times with 70% ethanol followed by rinsing five times with sterile water. Surface sterile seeds were soaked (seed biopriming) in the cell suspension of each rhizobacterial isolate according to the treatment, and were then incubated in the dark at 25 °C for 24 h. Bio-primed seeds were then placed at an equal distance on sterilized wet tissue paper in a petri dish (20 seeds per plate) using sterile forceps (five replicates per treatment) and kept in the plant growth chamber in the dark at 25 °C. Four days after germination, uniform seedlings were selected and transplanted into the growth pouch containing modified Hoagland nutrient solution prepared according to Premsuriya et al. (2017) and Nawara et al. (2020) [47,48]. Then, the cell suspensions (~10^6^ CFU·mL^−1^) were inoculated to the seedlings according to the treatments. Rice seedlings were initially irrigated with 1⁄4 strength Hoagland solution for 5 days, and the solution was replaced twice during this period. Then, the seedlings were irrigated with 1⁄2 strength Hoagland solution for 2 days. After that, the irrigation medium was changed to a full-strength Hoagland solution [49] supplemented with four salinity levels (0, 50, 100, and 150 mM NaCl). The average EC values of an irrigation medium were 2.06, 7.69, 13.78, and 19.51 dS·m^−1^, respectively. The full-strength Hoagland solution was filled twice a week to maintain the volume of an irrigation medium. The uninoculated (controls) and inoculated seedlings were grown in a climate-controlled room (12:12 light–dark photoperiod at 25 ± 3 °C under a light level of approximately 5.8 klux). The rice seedlings from each pouch were harvested at 30 days after transplanting. Leaves of KDML105 seedlings from each treatment (three replications) were used to determine the 2AP level.

### 2.4. Quantification of 2-Acetyl-1-pyrroline in KDML105 Rice Leaves

Rice leaves were dried in an oven at 60 °C for 3 days until the moisture content was reduced to 14% before being sent to Salana Organic Village (Social Enterprise) Co., Ltd., Nakhon Pathom, Thailand, for the determination of the 2AP content. The analysis of 2AP was made by static headspace gas chromatography coupled with a nitrogen/phosphorus detector (SHS-GC–NPD) by the method of [50].

### 2.5. Survival of Rhizobacterial Isolates

This experiment was conducted to evaluate the effect of different concentrations of NaCl on the population of the rhizobacterial isolates 30 days after transplanting. The presence of the PGPR isolates in the fresh rice roots was evaluated by microscopic analysis. The initial population of all isolates inoculating to the rice seedlings at transplanting time, was approximately 10^6^ CFU·mL^−1^ (OD_600_~0.2). One milliliter of sample from each treatment with four NaCl levels (0, 50, 100, and 150 mM NaCl) was taken after the end of the experiment. Serial dilutions were prepared, and 0.1 mL aliquots (10^−3^–10^−5^) were spread on nutrient agar (NA). The experiment was carried out with three replicates for each treatment. The survival of rhizobacterial isolates under salt-stress conditions was determined by observing the number of viable cells after exposure to NaCl. After incubation at 37 °C for 72 h, colonies that appeared on NA plates were counted. The number of colonies of NaCl-stressed rhizobacteria was compared with that of untreated rhizobacteria (without NaCl) and presented as the percentage survival using the following equation:

(1) $$\begin{array}{c} \%\;\mathrm{Survival}\;=\frac{\mathrm{CFU}\;\left(\mathrm{with}\;\mathrm{NaCl}\right)}{\mathrm{CFU}\;\left(\mathrm{without}\;\mathrm{NaCl}\right)}\times 100,\\ \mathrm{CFU}\;=\;\mathrm{colony}\;\mathrm{forming}\;\mathrm{unit}. \end{array}\;$$

### 2.6. Microscopic Analysis of Rhizobacterial Isolates

To confirm the colonization of the inoculated *Sinomonas* sp. ORF15-23 and *Bacillus* sp. strain CRF17-18 in the KDML105 rice roots under different NaCl concentrations, the microstructure of the rice roots from all treatments was examined under a scanning electron microscope (SEM) using lateral root samples of all the inoculated isolates. The roots were cut into 1 cm segments and were prefixed in 4% glutaraldehyde solution overnight and washed with 0.1 M sodium cacodylate buffer three times for 30 min each. Osmium tetraoxide buffer (1%) was used for post fixation. After a series of dehydration steps in acetone, the samples were dried in a critical point dryer and mounted on aluminum stubs, sputter-coated with gold, and viewed under low-vacuum scanning electron microscope (JEOL, model JSM5910LV, Tokyo, Japan) at Faculty of Science, Chiang Mai University.

### 2.7. Statistical Analysis

Two-way ANOVA together with Turkey’s HSD at the 1% probability level [51] was applied for analyzing collected data using Statistix 9 (Analytical Software, Inc., Tallahassee, FL, USA). 

## 3. Results

### 3.1. 2-Acetyl-1-pyrroline Production Potential of Rhizobacterial Isolates

**Figure 1:**
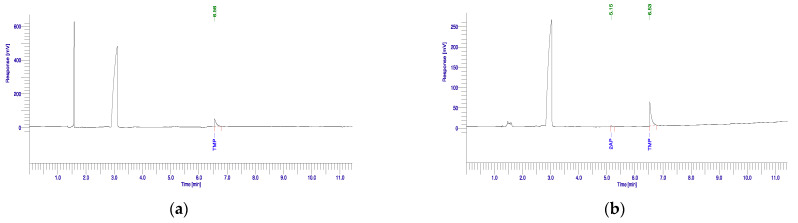
Chromatograms of GC–MS analysis of 2AP production for control nutrient broth without PGPR isolates (**a**) and *Sinomonas* sp. ORF15-23 (**b**).

The presence of 2AP was detected in all the culture liquid samples from the rhizobacterial isolates by sniffing. However, when the extracts from each culture liquid were examined for 2AP using a GC–MS, seven out of nine rhizobacterial isolates showed a peak with the same retention time as the 2AP standard. No peak was detected for the negative control (Figure 1a), as well as for strains CRF5-8 and *Bacillus* sp. CRF17-18. Significant differences (*p* ≤ 0.001) in 2AP production existed among the tested isolates, and the 2AP production is summarized in Table 2. The maximum 2AP production was recorded in *Sinomonas* sp. ORF15-23 (372.8 μg·kg^−1^, Figure 1b), followed by *Enterobacter* sp. ORF10-12, *Burkholderia* sp. CRF16-3, and *Micrococcus* sp. ORF15-19 with values of 330.2, 254.9, and 254.0 μg·kg^−1^, respectively.

### 3.2. Effect of Rhizobacterial Isolate Inoculation on the 2AP Level of KDML105 Rice Seedlings

The inoculation effects of nine rhizobacterial isolates were evaluated in *O. sativa* KDML105 rice seedlings under normal (0 mM NaCl) and saline conditions (50, 100, and 150 mM NaCl). Uninoculated seedlings at 0, 50, 100, and 150 mM NaCl were considered as control-0, control-50, control-100, and control-150, respectively. The term ‘control’ was used for the uninoculated seedling treatments of all salinity levels. These abbreviations are used throughout this publication. Statistical analysis showed a significant interaction (*p* ≤ 0.01) between PGPR isolates and NaCl concentrations for all the study variables (Table 3).

**Figure 2:**
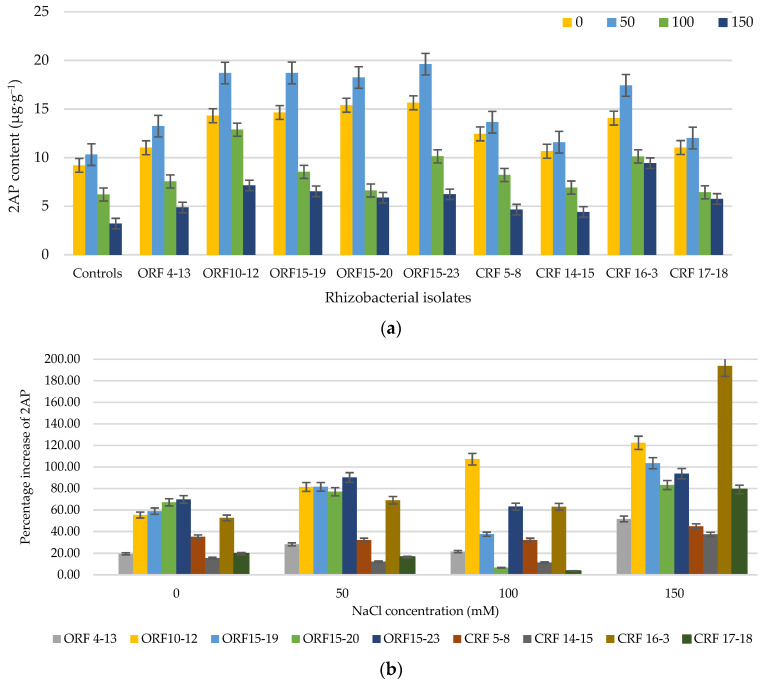
2-Acetyl-1-pyrroline (2AP) content in fresh KDML105 rice leaves (**a**), and percentage increase in 2AP (**b**) at 30 days after transplanting under saline conditions (0, 50, 100, and 150 mM NaCl).

The 2AP level in rice is widely used as one important indicator which reflects the high quality of aromatic rice. In the present study, the inoculation of PGPR isolates clearly enhanced the 2AP level in the leaves of KDML105 seedlings under both normal (0 mM NaCl) and saline conditions (50, 100, and 150 mM NaCl) (Figure 2a). Two-way ANOVA indicated that PGPR isolates and salinity effects on the 2AP content of rice were significant (*p* < 0.001). Under normal conditions, the application of PGPR isolates significantly increased the 2AP content of the fresh rice leaves in the range of 15.6–69.8% (10.65–15.64 µg·kg^−1^) as compared to control-0 (9.21 µg·kg^−1^) (Figure 2a).

Interestingly, under salinity stress, inoculation of the PGPR isolates also significantly increased the 2AP content in rice leaves in the range of 3.5–193.7% (4.41–19.61 µg·kg^−1^) as compared to each respective control. Among all the tested strains, the maximum 2AP content was observed upon inoculation of *Sinomonas* sp. ORF15-23 at 50 mM NaCl (19.6 µg·kg^−1^) with an increase of 90.20% as compared to control-50 (10.3 µg·kg^−1^) and *Burkholderia* sp. CRF16-3 at 150 mM NaCl (9.4 µg·kg^−1^) with a 193.7% increase as compared to control-150 (3.2 µg·kg^−1^). The 2AP content of all treatments increased with the increase in NaCl concentration up to 50 mM; however, beyond 50 mM NaCl, (100 and 150 mM NaCl), the 2AP content decreased with the increase in NaCl concentration. At 100 and 150 mM NaCl, strains ORF10-12 and CRF16-3 gave the highest 2AP levels with values of 12.87 and 9.43 µg·kg^−1^, equivalent to 107.2% and 193.7% increases, as compared to control-100 and control-150, respectively (Figure 2a,b).

### 3.3. Effect of Different NaCl Concentrations on the Survival of Rhizobacterial Isolates at 30 Days after Inoculation

Nine isolates were tested for their ability to survive under different concentrations of NaCl (0, 50, 100, and 150 mM NaCl) in Hoagland solution, 30 days after inoculation into the rice seedlings. In this study, we investigated the growth of rhizobacteria isolates in NA media for 72 h, after incubation at 37 °C, to reach the log phase of bacterial growth. The number of CFU of all the rhizobacterial isolates showed a decreasing trend with an increase in salt concentration (Table 4).

**Figure 3:**
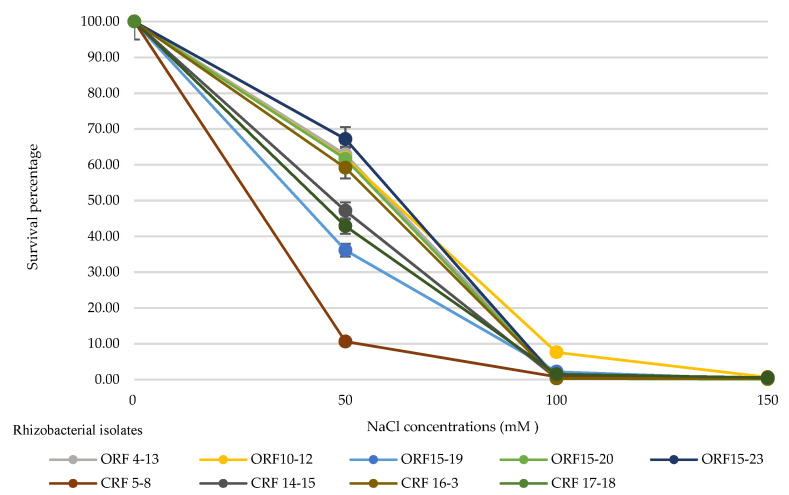
Survival of rhizobacterial isolates under different concentrations of NaCl in Hoagland’s solutions after 30 days of inoculation. The error bars represent the standard deviation of measurements for three replications.

Figure 3 shows the percentage of surviving bacteria, demonstrating a significant difference in salt tolerance ability between isolates cultured in Hoagland’s solutions containing 0–150 mM NaCl. In general, each rhizobacterial isolate was able to tolerate all tested NaCl levels with the remaining number of approximately 10^6^ CFU·mL^−1^ at 150 mM NaCl (Table 4). The growth of rhizobacterial isolates was slightly decreased from 0 to 50 mM NaCl but sharply decreased thereafter. In order to evaluate the relationship between salt-stress levels and the survival of the rhizobacterial strains, the number of treated rhizobacteria (under NaCl stress) was compared with that of untreated rhizobacteria (without NaCl) and presented as the survival percentage. The survival rate showed a decreasing trend with an increase in salt concentration. At 50 mM NaCl, the survival percentage was 36.1–67.2%, while, at 100 and 150 mM NaCl, these values were 0.3–7.6% and 0.1–0.7%, respectively (Figure 3). *Sinomonas* sp. isolate ORF15-23 showed the highest cell number at most NaCl levels (Table 4).

### 3.4. Microscopic Observations of Root Colonization

**Figure 4:**
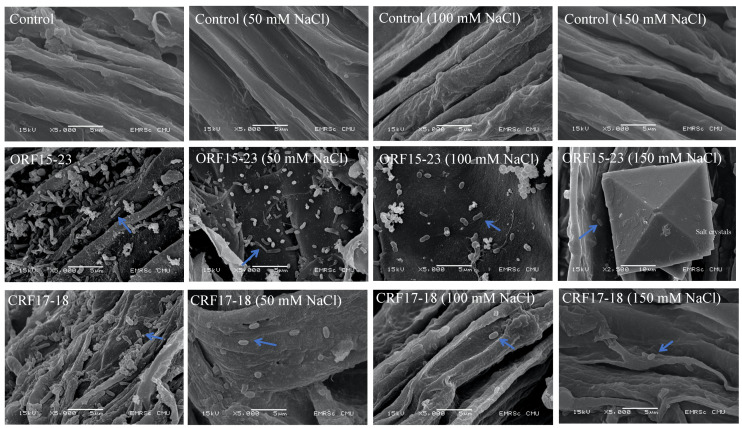
Root colonization of *Sinomonas* sp. strain ORF15-23 and *Bacillus* sp. strain CRF17-18 in KDML105 seedlings examined with a field-emission scanning electron microscope (scale bar, 5000× magnification). The figures are representative of three root samples per treatment.

The potential of rice root colonization by the different rhizobacterial isolates was determined by scanning electron microscopy (SEM). The SEM images showed, as expected, no root colonization in the uninoculated controls seedlings at 0, 50, 100, and 150 mM NaCl (Figure 4). Dense root colonization by all nine tested rhizobacterial isolates was clearly observed in inoculated seedlings (data not shown). However, the colonization density declined with the increasing salinity level, and no colonization was observed at 150 mM NaCl except for the seedling roots inoculated with *Sinomonas* sp. isolate ORF15-23 and *Bacillus* sp. strain CRF17-18. In addition, the inoculated roots of both non-stressed and salinity-stressed seedlings were colonized by *Sinomonas* sp. strain ORF15-23 and *Bacillus* sp. Strain CRF17-18 (Figure 4).

## 4. Discussion

2-Acetyl-1-pyrroline (2AP), a nitrogen-containing compound, is the major compound responsible for the unique aromatic characteristic in rice including the Thai jasmine rice/Thai aromatic rice KDML105 cultivar [52,53,54,55]. This aromatic compound has also been reported to be produced by a wide range of microorganisms, including plant-growth-promoting rhizobacteria (PGPR) [5,27,29]. A previous study by Deshmukh et al. [29] reported that rice rhizobacteria could produce the 2AP compound with values ranging from 6.53–119.3 µg·kg^−1^. Higher 2AP production was observed in rhizobacteria obtained from the rhizosphere of aromatic rice compared to nonaromatic rice. All isolates from our study which were obtained from the aromatic rice rhizosphere showed a higher 2AP content than this earlier report. A similar observation was reported by Dhondge et al. [5], who found the maximum 2AP production in *E. hormaechei* and *L. xylanilyticus* isolated from the aromatic rice rhizosphere. In this study, for the first time, we provide evidence that members of the high-GC Gram-positive bacteria *Micrococcus* and *Sinomonas* are able to produce the 2AP compound. Members of the genera *Bacillus* (low-GC Gram-positive bacteria) and *Burkholderia* (Gram-negative bacteria) are also added to the list of 2AP-producing bacteria. This observation supports the view that the ability to produce the 2AP compound is widely distributed among bacteria. Our finding and previous reports strongly suggest that the rhizosphere of aromatic rice is a good source for 2AP producing rhizobacteria. Since a gradual decrease in the 2AP level in KDML105 rice grains is eminent due to excessive soil salinity driven by chemical inputs and climate change, this 2AP-producing PGPR might provide a sustainable alternative solution to cope with the aroma deterioration problems.

In the present study, all PGPR isolates from the ORF system produced 2AP in the range of 30.91–372.76 µg·kg^−1^. However, 2AP production was found in only half of the tested PGPR isolates from the CRF system (0–254.95 µg·kg^−1^). It was interesting to note that, on average, PGPR isolates obtained from the ORF system gave higher 2AP production (216.98 µg·kg^−1^) in the culture medium than PGPR isolates obtained from the CRF system (133.48 µg·kg^−1^). In addition, only one CRF isolate (*Burkholderia* sp. CRF16-3) showed high 2AP production (254.95 µg·kg^−1^), while the highest 2AP value (372.76 µg·kg^−1^) was obtained from an ORF isolate (*Sinomonas* sp. ORF15-23). These observations support the view that agricultural practices play a role in aromatic level of aromatic rice. Our previous study suggested that the high soil bacterial population and total soil nitrogen from an ORF system of the TKR area were responsible for the 3.5-fold higher 2AP content of KDML105 rice grains in this system than the CRF [30]. Appropriate soil environments and sufficient N supply in the ORF system might promote a higher 2AP production by PGPR isolated from this system. Although PGPR have been extensively investigated, few studies have correlated agricultural practices with the plant-growth-promoting capacity of rhizobacteria isolated from different systems, e.g., organic vs. conventional farming systems. This aspect requires further research effort before a definitive conclusion can be reached.

The high 2AP level in the KDML105 rice leaves led to a high 2AP level in the rice grains grown in clay and sandy soil [56]. Therefore, in the present study, it was expected that the enhancement of the 2AP level in the leaves of KDML105 rice seedlings by ST-PGPR inoculation might lead to high 2AP in the rice grains. A previous study revealed that secondary metabolites including 2AP are mostly produced in rice plant during the booting stage [57]. In addition, previous studies suggested that the 2AP was translocated from the leaves to the grain during later stages of rice growth [58,59,60]. The 2AP content in rice continuously increased from the seedling stage to the maximum level around the booting stage and the enhancement of 2AP content at or after booting stage was reported to increase 2AP accumulation in mature grains [59]. Therefore, the enhancement of 2AP by ST-PGPR inoculation during the seedling stage might also increase the 2AP level in mature grains similar to inoculation at the booting stage. The determination of 2AP level in leaves might be useful for prediction of the 2AP level in the rice grains.

The inoculated aromatic rice variety Basmati-370 had around a 1.14–1.41-fold higher 2AP level than the uninoculated control [29]. The highest 2AP-producing strain provided the highest 2AP in the aromatic rice, while the 2AP-nonproducing strains had no significant effect on the 2AP contents of Basmati-370. Our results indicated that the inoculation of 2AP producing PGPR markedly enhanced the 2AP level in the rice seedling leaves under both normal (15.64–69.82% increase) and saline conditions (3.54–193.77% increase). The inoculation of 2AP-producing *Enterobacter hormaechei* (AM122) and *Lysinibacillus xylanilyticus* (DB25) in consortium resulted in a significant improvement in 2AP content of Basmati rice over mono inoculation and control [5]. Interestingly, the highest 2AP-producing PGPR, *Sinomonas* sp. ORF15-23, provided the maximum 2AP content in the seedling leaves at 50 mM NaCl (19.6 µg·kg^−1^), with a percentage increase of 90.20% as compared to control-50. It was observed that, on average, the 2AP level of the inoculated seedling leaves showed much higher values than those of the uninoculated controls at all tested NaCl concentrations. However, as compared to each respective control, the 2AP value of the inoculated seedling leaves showed a sharp increase within a certain range of NaCl concentrations (0–50 mM NaCl with an EC value of 2.06 to 7.69 dS·m^−1^, respectively). These 2AP values slightly increased beyond this NaCl range (100 and 150 mM NaCl with EC values of 13.78 and 19.51 dS·m^−1^, respectively). According to the salinity tolerance, rice is considered a salt-sensitive crop, and the rice seedling and reproductive stages are regarded as the most sensitive periods to salt stress, in which the growth and yield of rice are greatly affected by salinity with a threshold EC value of 3 dS·m^−1^ for most cultivated varieties [14,61]. Various studies revealed that the rice biomass, grain yield, and quality were drastically affected by salinity beyond 8 dS·m^−^^1^ [17,18,62]. Although, the 2AP content in the seedling leaves at 150 mM NaCl was much lower than in those at 50 mM NaCl, the percentage increase was higher. This phenomenon suggests that the maximum percentage increase in 2AP production at 150 mM was mainly due to an excessive salinity stress. As compared to the uninoculated control, our results clearly indicate that the application of PGPR isolates could effectively enhanced the 2AP synthesis in rice seedling leaves when the EC value was <8 dS·m^−1^; however, beyond this EC value, the PGPR isolates showed only slight upregulation of the 2AP synthesis. This phenomenon implies that the highly positive interaction between the PGPR isolates and the seedlings for rice 2AP synthesis could be expected when the EC value of the medium was less than the threshold value (~8 dS·m^−1^).

The key aspects dominating the success of PGPR inoculation are their survival number in the rhizosphere environment and the establishment of the introduced bacteria in the plant roots. In the present study, the number of PGPR isolates slightly decreased from 0 to 50 mM NaCl but sharply decreased thereafter (>50 mM NaCl). Although the population decreased beyond 50 mM NaCl, each isolate remained around 10^6^ CFU·mL^−1^ at 150 mM NaCl. Therefore, these PGPR isolates could be considered as salt-tolerant PGPR (ST-PGPR). Deshmukh et al. concluded that the optimal number of rhizobacterial isolates which showed a highly positive influence on rice 2AP level is 10^5^–10^6^ CFU·mL^−^^1^, whereas 10^2^–10^4^ CFU·mL^−1^ showed less influence on the 2AP level [29]. This observation suggested that the ST-PGPR tested in this study could probably survive under salt-stressed soil, such as the TKR region. As these strains were reported to solubilize P and K, as well as produce siderophore, they could be applied for the enhancement of rice growth as well as 2AP level in the KDML105 rice grains [30]. A study showed that *Streptomyces* isolate S3, which produces IAA and siderophore and solubilizes phosphate, enhanced the growth of KDML105 rice seedlings under drought stress, as phosphorus and iron are essential for plant growth [7]. Therefore, the nine strains obtained in this study have high potential application in the rice field. Members of the genus *Sinomonas* are Gram-positive bacteria in the phylum Actinobacteria [63]. Currently, there are 10 validly described species on the List of Prokaryotic names with Standing in Nomenclature (LSPN) [https://www.bacterio.net/genus/sinomonas (accessed date 13 September 2021)] [64]. A search through the Online Risk Group Database provided by ABSA International (the Association for Biosafety and Biosecurity) found no record regarding the risk of members of the genus *Sinomonas* as animal, human, or plant pathogens or select agents by both the Center for Disease Control (CDC) and the United States Department of Agriculture (USDA). In addition, to our best knowledge, there are no publications reporting the risk of *Sinomonas* species toward the environment or human health.

The adhesion of bacteria to the seeds is the first step in rhizobacteria–plant interactions for successful colonization [65,66]. Colonization of beneficial bacteria is of central importance for growth-promoting activity [65]. All PGPR in this study exhibited root colonization ability in KDML105 seedlings as examined by SEM analysis. *Sinomonas* sp. strain ORF15-23 was able to colonize both non-stressed and salinity-stressed seedlings of KDML105 rice. The SEM results of Dhondge et al. [5] revealed that cells of *L. xylanilyticus* and *E. hormaechei* were consistently distributed and adhered to the root surface of inoculated seedlings in clusters as microcolonies. The formation of a microcolony by rhizobacteria on the surface of the root indicates successful colonization [67]. Exposure to excessive salinity was found to decrease maize and wheat root attachment by *Azospirillum brasilense* [68]. This finding is in accordance with the results of the present study, which showed that the colonization density declined with an increase in salinity level, clearly indicating a negative impact of salt stress on rhizobacterial population (Figure 4). However, the most salt-tolerant isolate, *Sinomonas* sp. ORF15-23, maintained its high density upon root attachment at all NaCl levels, and this might be the reason for its greatest ability in promoting the 2AP level of rice seedling leaves.

## 5. Conclusions

In conclusion, the present investigation revealed that most of the tested PGPR isolates are able to produce 2AP (a key volatile aroma compound in Thai jasmine rice KDML105) in liquid medium in vitro. The inoculation of these PGPR isolates, particularly *Sinomonas* sp. strain ORF15-23, markedly enhanced the 2AP level in the leaves of rice seedlings under salinity stress up to 150 mM, which could lead to a high 2AP level in the rice grains, thus promoting the economic value of KDML105 aromatic rice. In addition, all PGPR isolates are considered salt-tolerant PGPR, as they survived in 150 mM NaCl at high cell density. *Sinomonas* sp. strain ORF15-23 was also able to colonize the roots of both non-stressed and salinity-stressed seedlings. Our findings clearly indicated that 2AP-producing rhizosphere PGPR isolates are potential candidate for promoting the growth and aromatic compound level of Thai jasmine rice KDML105 under salinity stress. Further investigation under field conditions is needed for the development of these promising PGPR isolatess, especially *Sinomonas* sp. strain ORF15-23, as a bioinoculant for KDML105 rice cultivation in salt-affected areas.

## Figures and Tables

**Table 1:** Phosphate and potassium solubilization, as well as siderophore production, by the nine selected KDML105 rice rhizobacterial isolates.

Farming Practice	Rhizobacterial Isolate	Genus	Phosphate (Ca-P) Solubilization	Potassium (K-Feldspar) Solubilization	Siderophore Production
			Soluble P	Soluble K	Hydroxamate-Type
			(mg·L^−1^)	(mg·L^−1^)	(µmol·L^−1^)
*Organic farming*	ORF4-13	*Sinomonas* sp.	35.6	37.3	110.8
	ORF10-12	*Enterobacter* sp.	31.6	39.4	162.5
	ORF15-19	*Micrococcus* sp.	11.6	34.6	64.2
	ORF15-20	*Micrococcus* sp.	25.0	30.7	83.3
	ORF15-23	*Sinomonas* sp.	20.9	49.5	22.5
*Conventional farming*	CRF5-8	unidentified	31.6	44.8	2.5
	CRF14-15	*Sinomonas* sp.	36.6	38.6	1.7
	CRF16-3	*Burkholderia* sp.	17.5	43.0	618.3
	CRF17-18	*Bacillus* sp.	35.0	28.7	0.5

Adapted from Chinachanta and Shutsrirung [45].

**Table 2:** 2-Acetyl-1-pyrroline (2AP) production by selected rhizobacterial isolates.

Farming Practice	Rhizobacterial Isolate	2AP Production (μg·kg^−1^)
	Uninoculated control	ND ^1^
*Organic farming*	ORF4-13	30.91 ± 0.43 e
	ORF10-12	330.24 ± 1.70 b
	ORF15-19	254.04 ± 3.89 c
	ORF15-20	96.94 ± 1.49 d
	ORF15-23	372.76 ± 7.99 a
		*Average* 216.98
**Farming Practice**	**Rhizobacterial Isolate**	**2AP Production (μg·kg^−1^)**
*Conventional farming*	CRF5-8	ND
	CRF14-15	12.00 ± 1.53 f
	CRF16-3	254.95 ± 2.48 c
	CRF17-18	ND
		*Average* 133.48
	F-test	**
	LSD_(0.05)_	6.37
	CV%	1.88

^1^ ND = not detectable; ** significant at the 0.001 probability level.

**Table 3:** Significance level and Turkey’s HSD values for studied variables.

Source of Variance	The 2AP Content
PGPR isolates (A)	**
NaCl concentration (B)	*
A × B	**
Turkey’s HSD _(0.01)_ for (A × B)	1.0995
CV%	3.6756

** Significant at the 0.01 probability level; * significant at the 0.05 probability level.

**Table 4:** Number of viable rhizobacteria cells under different NaCl concentrations in Hoagland solution at 30 days after inoculation.

Rhizobacteria Isolates	Number of Viable Cells (CFU·mL^−1^)			
	NaCl Concentrations (mM)			
	0	50	100	150
Organic rice farming				
ORF4-13	5.8 × 10^8^	3.7 × 10^8^	6.0 × 10^6^	2.0 × 10^6^
ORF10-12	3.5 × 10^8^	2.2 × 10^8^	2.7 × 10^7^	2.3 × 10^6^
ORF15-19	2.0 × 10^9^	7.2 × 10^8^	4.3 × 10^7^	2.3 × 10^6^
ORF15-20	1.4 × 10^9^	8.8 × 10^8^	4.3 × 10^6^	1.0 × 10^6^
ORF15-23	2.2 × 10^9^	1.5 × 10^9^	6.1 × 10^6^	4.8 × 10^6^
Conventional rice farming				
CRF5-8	8.0 × 10^8^	8.5 × 10^7^	6.1 × 10^6^	4.8 × 10^6^
CRF14-15	1.2 × 10^9^	5.5 × 10^8^	4.5 × 10^6^	3.1 × 10^6^
CRF16-3	1.2 × 10^9^	7.0 × 10^8^	3.3 × 10^6^	3.0 × 10^6^
CRF17-18	4.7 × 10^8^	2.0 × 10^8^	7.0 × 10^6^	2.5 × 10^6^

## Data Availability

Data sharing does not apply to this article as no datasets were generated or analyzed during the current study.
